# Co-transmitting neurons in the lateral septal nucleus exhibit features of neurotransmitter switching

**DOI:** 10.1016/j.ibneur.2022.05.003

**Published:** 2022-05-12

**Authors:** Patrick J. Hunt, Mikhail Kochukov, Brandon T. Pekarek, Benjamin D.W. Belfort, Juan M. Romero, Jessica L. Swanson, Benjamin R. Arenkiel

**Affiliations:** a Genetics and Genomics Program, Baylor College of Medicine, 1 Baylor Plaza, Houston, TX, USA; b Department of Molecular and Human Genetics, Baylor College of Medicine, 1 Baylor Plaza, Houston, TX, USA; c Medical Scientist Training Program, Baylor College of Medicine, 1 Baylor Plaza, Houston, TX, USA; d Neuroscience Graduate Program, Baylor College of Medicine, 1 Baylor Plaza, Houston, TX, USA; e Department of Neuroscience, Baylor College of Medicine, 1 Baylor Plaza, Houston, TX, USA; f Jan and Dan Duncan Neurological Research Institute at Texas Children's Hospital, 1250 Moursund Street, Houston, TX 77030, USA

**Keywords:** Acetylcholine, Co-transmission, GABA, Lateral Septal Nucleus, Neurotransmitter, Neurotransmitter switching

## Abstract

The lateral septal nucleus (LSN) is a highly interconnected region of the central brain whose activity regulates widespread circuitry. As such, the mechanisms that govern neuronal activity within the LSN have far-reaching implications on numerous brain-wide nuclei, circuits, and behaviors. We found that GABAergic neurons within the LSN express markers that mediate the release of acetylcholine (ACh). Moreover, we show that these vGAT^LSN^ neurons release both GABA and ACh onto local glutamatergic LSN neurons. Using both short-term and long-term neuronal labeling techniques we observed expression of the cholinergic neuron marker Choline Acetyltransferase (ChAT) in vGAT^LSN^ neurons. These findings provide evidence of cholinergic neurotransmission from vGAT^LSN^ neurons, and provide an impetus to examine dynamic co-neurotransmission changes as a potential mechanism that contributes to neuronal and circuit-wide plasticity within the LSN.

## Introduction

Co-transmission of multiple neurotransmitters from the same neuron provides an adaptable means to regulate neural circuit activity. This is especially true in cases in which both an excitatory and an inhibitory neurotransmitter are released from the same neuron. Maintaining excitatory/inhibitory balance from multiple sources is crucial for proper functioning of neural circuits (Tatti et al., 2017). Consequently, when multiple different neurotransmitters are released onto the same postsynaptic cell, neurons and their respective circuits are afforded an increased spatiotemporal precision in regulating postsynaptic neuronal activity (Root et al., 2014, Shabel et al., 2014). This is opposed to when each neurotransmitter is released onto different postsynaptic neurons, which provides co-releasing neurons the unique ability to activate certain nodes of the circuit while simultaneously inhibiting others (Granger et al., 2016). The flexibility of such a signaling paradigm enables an expansion of signaling diversity, while also facilitating the ability of neural circuits to quickly respond to changes in their environment. Mechanisms that drive neural circuit plasticity provide even further flexibility, allowing circuits and the neurons within them to adapt to changing environmental cues. The co-transmission of acetylcholine (ACh) and GABA has been well documented within multiple nuclei and brain regions (Lee et al., 2010, Saunders et al., 2015b, Saunders et al., 2015a, Granger et al., 2016, Granger et al., 2020, Case et al., 2017, Takács et al., 2018). However, whether there is a continuous capability for plasticity within these circuits into adulthood, and the role that this plasticity plays in co-transmission is not well understood.

In response to activity-dependent cues, certain populations of neurons alter transcription or release of new or different neurotransmitters (Spitzer, 2015, Spitzer, 2017; Li and Spitzer, 2020). Neurotransmitter switching often changes inhibitory neurons to excitatory neurons, or vice-versa, allowing key neurons within a circuit the ability to change the sign of their effect. Cellular state plasticity is capable of altering behavior (Dulcis et al., 2013, Spitzer, 2015, Spitzer, 2017) through transcription-based changes in ACh and GABA release (Li and Spitzer, 2020). Though neurotransmitter switching might help explain the co-transmission of multiple neurotransmitters from the same neuron (Spitzer, 2015, Granger et al., 2016), it is not known whether neurotransmitter switching affects co-transmission events recorded throughout the brain. Moreover, only a limited number of brain regions have been identified in which dynamic neurotransmitter switching occurs (Spitzer, 2017).

The Lateral Septal Nucleus (LSN) is a centrally located and highly interconnected nucleus within the forebrain that lies just medial to the lateral ventricles. Traditionally this nucleus has been implicated in the regulation of anxiety and aggression (Albert and Richmond, 1976, Sheehan et al., 2004, Singewald et al., 2011, Leroy et al., 2018), however recent reports have uncovered previously overlooked roles for the LSN (Sweeney and Yang, 2015, Sweeney and Yang, 2016). Studies of the LSN have primarily focused on GABAergic (Zhao et al., 2013, Sweeney and Yang, 2016, Leroy et al., 2018, van der Veldt et al., 2021) and glutamatergic neurons (Kocsis et al., 2003, Lin et al., 2003, Sheehan et al., 2004). However, much remains unknown regarding other cell types within the LSN, including their mode of neurotransmission, their projection targets, and how these features dictate behavioral regulation. Though LSN neurons develop from the same neurogenic niche as the cholinergic neurons of the medial septum (Verney et al., 1987, Sheehan et al., 2004), cholinergic neurons within the LSN have not been previously investigated (Ichikawa et al., 1997, Lein et al., 2007, Rossi et al., 2011, Saunders et al., 2015a).

Here we present evidence of cholinergic transmission from the LSN. Moreover, we identified ACh/GABA co-expressing and co-transmitting neurons within the LSN that exhibit features of neurotransmitter switching. We find that these co-transmitting neurons project onto local glutamatergic neurons within the LSN, thereby regulating local neural circuit activity.

## Experimental procedures

### Animals

All mice in this study were treated in compliance with US Department of Health and Human Services and Baylor College of Medicine IACUC guidelines. Both male and female mice were used in analyses. Mice were between 3 and 5 months of age for surgeries, and between 3 and 18 months of age for experiments. All animals were maintained on a 12 hr light-dark cycle. Mice were group housed. ChAT-Cre (ChAT^tm1(cre)Lowl^; Stock No. 031661), vGAT-Flp (Slc32a1^tm1.1(flpo)Hze^; Stock No. 029591), vGAT-Cre (Slc32a1^tm2(cre)Lowl^; Stock No. 028862), and vGlut2-Flp (Slc17a6^tm1.1(flpo)Hze^; Stock No. 030212) mice were originally purchased and are available from Jackson Laboratories. Genotyping for Cre was done using the following primers: forward 5’-GCATTACCGGTCGATGCAACGAGTGATGAG-3’ and reverse 5’-GAGTGAACGAACCTGGTCGAAATCAGTGCG-3’. Genotyping for vGAT-Flp was done using the following primers: wildtype forward 5’-GTCTGCGTTTCTGTCGTCCT-3’, wildtype reverse 5’-CTCAAGGTCAAGTTTCCAAGC-3’, mutant forward 5’-TGCATCGCATTGTCTGAGTAG-3’, and mutant reverse 5’-GACAGCCGTGAACAGAAGG-3’. Genotyping for vGlut2-Flp was done using the following primers: common forward 5’-GAAACGGGGGACATCACTC-3’, wildtype reverse 5’-GGAATCTCATGGTCTGTTTTG-3’, and mutant reverse 5’-ACACCGGCCTTATTCCAAG-3’.

### Stereotaxic injections and viral constructs

For all stereotaxic injections, mice were anesthetized and maintained under anesthesia using vaporized isoflurane with O2. All injections were performed using a stereotaxic apparatus synced to Angle Two software for coordinate guidelines. The LSN was targeted through bilateral injections (from bregma AP = 1.00 mm, DV = −3.30 mm, and ML = +0.30 mm). Viruses used in experiments include: AAV-Ef1a-DIO-mVenus-WPRE-hGHpA Serotype DJ8, AAV-Ef1a-fDIO-mRuby2-WPRE-hGHpA Serotype DJ8, AAV-nEF-COn/FOn-hChR2(H134R)-EYFP-WPRE-hGHpA, serotype DJ8, AAV-Ef1a-DIO-ChR2-EYFP-WPRE-hGHpA Serotype DJ8. For all viral experiments, 690 nL of virus or viral mixture (in cases in which two viruses are injected into the same region) was injected.

### Fluorescent in situ hybridization

All brain sections analyzed by FISH were prepared by the In Situ Hybridization core at the Jan and Dan Duncan Neurological Research Institute at Texas Children’s Hospital. ChAT RNA was stained for using the following probe: TTGCTTGGTGTGGAACAGTGCCGGTTCGGTGCGTAACAGCCCAGGAGAGCAGGTCGGCAGCTCTGCTACTCTGGATTAAGAATCGCTAGGATGCCTATCCTGGAAAAGGTCCCCCCAAAGATGCCTGTACAAGCTTCTAGCTGTGAGGAGGTGCTGGACTTACCTAAGTTGCCAGTGCCCCCACTGCAGCAAACCCTGGCCACCTACCTTCAGTGCATGCAACACCTGGTACCTGAAGAGCAGTTCAGGAAGAGCCAGGCCATTGTGAAGCGGTTTGGGGCCCCTGGTGGCCTGGGTGAGACCCTGCAGGAAAAGCTCTTGGAGAGACAGGAGAAGACAGCCAATTGGGTCTCTGAATACTGGCTGAATGACATGTATCTAAACAACCGCCTGGCCCTGCCAGTCAACTCTAGCCCTGCTGTGATCTTTGCTCGGCAGCACTTCCAAGACACCAATGACCAGCTAAGGTTTGCAGCCAGCCTCATCTCTGGTGTGCTTAGCTACAAGGCTCT (Lein et al., 2007). vGAT RNA was stained for using the following probe: GCCATTCAGGGCATGTTCGTGCTGGGCCTACCCTACGCCATCCTCCACGGCGGCTACCTGGGGTTGTTCCTCATCATCTTCGCCGCAGTGGTGTGCTGCTACACCGGCAAGATCCTCATCGCGTGCCTGTACGAGGAGAACGAAGACGGGGAGGTGGTGCGCGTGCGGGACTCGTATGTGGCCATAGCTAACGCATGCTGCGCTCCTCGATTCCCCACCCTGGGCGGCCGCGTGGTCAATGTGGCGCAGATCATCGAGCTGGTGATGACGTGTATCTTGTACGTCGTGGTGAGCGGCAACCTCATGTACAACAGTTTCCCGGGGCTGCCCGTGTCGCAGAAGTCCTGGTCCATCATAGCCACAGCGGTGCTGCTGCCCTGCGCCTTCCTGAAGAATCTCAAGGCCGTGTCCAAGTTCAGTCTGCTGTGTACGCTGGCCCACTTCGTCATCAACATCCTGGTCATCGCTTACTGTCTCTCTCGCGCGCGTGATTGGGCCTGGGAGAAGGTGAAGTTCTACATCGACGTCAAGAAGTTTCCCATCTCCATTGGCATCATCGTGTTCAGCTACACGTCGCAGATCTTCCTGCCCTCTCTCGAAGGCAACATGCAGCAGCCCAGCGAATTCCACTGCATGATGAACTGGACACACATCGCCGCCTGCGTGCTCAAGGGTCTCTTCGCGCTCGTCGCCTACCTCACCTGGGCCGACGAGACCAAGGAAGTCATCACGGATAACCTGCCCGGCTCCATCCGCGCCGTGGTCAACCTCTTCCTGGTGGCCAAGGCGCTGCTGTCCTATCCGTTGCCCTTCTTCGCGGCCGTCGAAGTGCTGGAGAAGTCTCTCTTCCAGGAAGGCAGTCGCGCCTTCTTCCCCGCCTGCTATGGAGGCGACGGTCGCCTTAAGTCCTGGGGGCTGACGCTGCGCTGCGCGCTGGTGGTCTTCACGCTGC (Lein et al., 2007). Adult mouse brains were collected and immediately embedded and frozen in Tissue-Tek OCT compound. All coronal sections were collected sequentially from the forebrain at 25 µm thickness. *In situ* hybridization was performed as previously described (Yaylaoglu et al., 2005). Stained sections containing the LSN were imaged using a Leica TCS SP8 STED microscope.

### Immunohistochemistry and microscopy

Animals were deeply anesthetized using isoflurane and were transcardially perfused with PBS followed by 4% PFA (diluted from 16% Paraformaldehyde EM Grade No. 15710 Electron Microscopy Sciences). Brains were dissected and post-fixed in 4% PFA overnight at 4 degrees Celsius. Brains were cryoprotected in 30% sucrose/PBS solution for 1 more day at 4 degrees Celsius. Brains were then embedded and frozen in O.C.T. (Fisher HealthCare No. 4585) and stored at − 80 degrees Celsius until sliced. Brains were sliced using a cryostat (Leica CM1860) in coronal 40 µm sections. For anti-ChAT immunohistochemistry, 40 µm free-floating sections were blocked for 1 hr at room temperature in 10% donkey serum blocking solution made in PBS-T (1X PBS, 1% Triton-X 100, pH 7.35). Sections were incubated for 14 days at 4 degrees Celsius at 1:500 dilution of block solution containing goat anti-ChAT primary antibody (Millipore AB144P). Sections were washed 5 times for 10 min in plain PBS-T. Sections were then incubated in secondary antibodies (1:500 donkey anti-goat Alexafluour-488) for 2 hr at room temperature. Sections were then washed 5 times at 10 min each in PBS-T. All sections were mounted using DAPI Fluoromount-G (SouthernBiotech, 0100–20). Sections from ChAT-Cre;vGAT-Flp;Tri-cistronic mice, and mice injected with AAV were washed with PBS 5 times for 10 min, blocked at 1 hr at room temperature in 10% donkey serum blocking solution, washed 5 times with PBS-T for 10 min, and then mounted with DAPI Fluoromount-G. Detection of fluorescent expression was performed using a Leica TCS SPE confocal microscope, or Leica TCS SP8 STED microscope.

### Electrophysiology

For all slice experiments, animals were anesthetized with isoflurane and perfused with cold artificial cerebrospinal fluid (ACSF) solution pH 7.35 mOsm 305–315 containing: 125 mM NaCl, 2.5 mM KCl, 1.25 mM NaH2PO4-H2O, 2 mM CaCl2, 1 mM MgCl2, 10 mM Glucose, 25 mM NaHCO3. Brains were rapidly removed and transferred into sucrose-based cutting solution pH 7.35 containing: 87 mM NaCl, 2.5 mM KCl, 1.25 mM NaH2PO4-H2O, 0.5 mM CaCl2, 7 mM MgCl2–6H2O, 13 mM Ascorbic Acid, 75 mM Sucrose, 10 mM Glucose, 25 mM NaHCO3 and continuously bubbled with 5% CO2/95% O2. 300 µm thick coronal brain slices were prepared using a Leica VT1200 vibratome and placed in recovery for 15 min at 37 degrees Celsius in 5% CO2/95% O2 bubbled ACSF solution. They were then gradually lowered to room temperature (25 degrees Celsius) and allowed to acclimate for at least 15 min before recording. For recording, slices were transferred into a recording chamber continuously perfused at 1–2 mL/min at 25 degrees Celsius. Neurons were identified by transmitted light DIC and fluorescent imaging (BX50WI, Olympus). Recordings were obtained using an Axon MultiClamp 700B amplifier digitized at 10 kHz (Axon Digidata 1440 A). Recording electrodes (3–5 megaohms) were fabricated from borosilicate glass microcapillaries (outer diameter, 1.5 mm) with a micropipette puller (Sutter Instruments). For voltage clamp recordings, internal solution containing the following was used: 120 mM Cs-methanesulfonate, 2 mM MgCl2–6H2O, 0.05 mM CaCl2, 6 mM CsCl, 20 mM HEPES, 0.2 mM EGTA, 10 mM phosphocreatine di(Na) salt, 4 mM ATP-Mg, 0.4 mM GTP-Na, pH 7.2 with CsOH, and 290–300 mOsm with CsMeSO4.

### Channelrhodopsin electrophysiology

Brain slices containing the LSN were prepared from 12 to 16 week-old animals that had been virally injected with AAV-DIO-ChR2-EYFP and AAV-fDIO-mRuby2 between 2 and 4 weeks prior to recording. Cells were patched using voltage clamp internal solution. Cells were recorded for a minimum of 10 sweeps for each pharmacological condition. Each sweep consisted of 2 s of recording, with an excitation duration of 1 ms of blue light (470 nm) exposure with 30 s between each sweep. ACSF with the following pharmacological concentrations in the slice chamber were used: 1 µM TTX (Tocris, 0.5 mM 4-AP (Tocris), 20 µM AP-5 (Tocris), 10 µM CNQX (Tocris).

### Quantification

The number of cells expressing viral, or FISH signal, as well as the amount of EYFP signal expressed following AAV-nEF-COn-FOn-ChR2-EYFP injections were quantified using the Imaris (Bitplane AG, Zürich, Switzerland) software.

### Contact for reagent and resource sharing

Further information and requests for resources and reagents should be directed to and will be fulfilled by the Lead Contact, Benjamin Arenkiel (Arenkiel@bcm.edu).

## Results

### LSN neurons co-express cholinergic and GABAergic markers

**Fig. 1:**
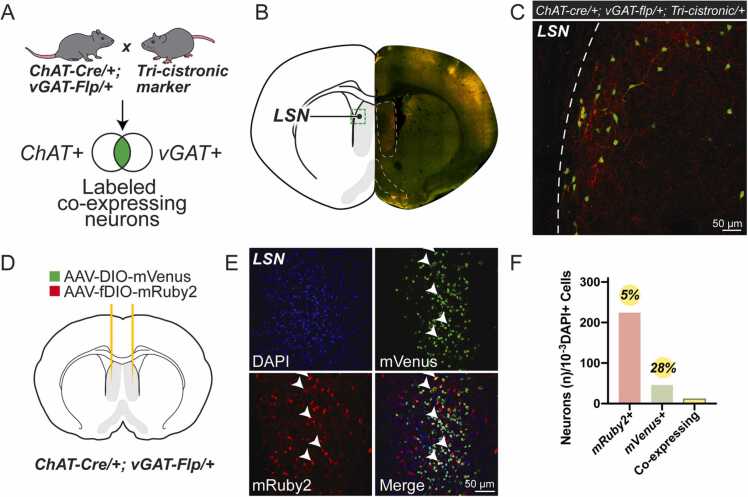
Genomic markers and viral labeling reveal ChAT/vGAT co-expressing population of neurons within the LSN. (A) Genomic or viral-based labeling of co-expressing neurons. (B) Location of the LSN. Green box indicates the location of the image shown in (C). Right, hemisphere of brain shown in (C). (C) ChAT/vGAT-positive neurons in the LSN labeled by tri-cistronic reporter. (D) Viral labeling of co-expressing LSN neurons. (E) Overlapping DIO-mVenus and fDIO-mRuby2 signals illustrating virally-targeted co-expressing neurons. (F) Quantified neurons within the LSN. Co-expressing neurons comprise 5% and 28% of vGAT-positive and ChAT-positive neurons, respectively. vGAT- and ChAT-positive neurons in each slice are normalized to the number of DAPI-positive nuclei counted in each slice. n = 28 images of the LSN from 1 animal.

To identify the distribution of ACh/GABA co-transmitting neurons throughout the brain, we first sought to label all neurons that express markers for the release of these two neurotransmitters. We identified the acetylcholine synthetic enzyme, ChAT, as well as the vesicular GABA transporter, vGAT, as reliable and faithful markers of cholinergic and GABAergic neurons respectively (Armstrong et al., 1983, Chaudhry et al., 1998, Rossi et al., 2011, Vong et al., 2011, Gizowski and Bourque, 2020, Graybuck et al., 2021). To identify putative co-transmitting neurons, we generated mice with three transgenic alleles to facilitate cell type-specific labeling. These included ChAT-Cre (JAX Stock No. 031661), vGAT-Flp (JAX Stock No. 029591), and a tri-cistronic fluorescent label that is Cre- and Flp-dependent (Supplementary Fig. 1A) (Lusk et al., 2022). In these animals, all neurons that are both ChAT-positive and vGAT-positive express the tri-cistronic allele, and are thus labeled with a nuclear BFP, a cytosolic GFP, and a membrane-bound tdTomato (Fig. 1A and Supplementary Fig. 1). From this cross, we identified tri-cistronic-positive neurons in the LSN (Fig. 1B-C), revealing a population of cholinergic and GABAergic co-expressing neurons in this area.

Certain neuronal populations switch the expression of neurotransmitter-related genes during development (Kotak et al., 1998, Nabekura et al., 2004, Spitzer, 2017). To confirm that these fluorescently labeled neurons are indeed co-expressing neurons rather than neurons that only expressed these markers at some point in development, we sought to verify the expression of cholinergic and GABAergic markers in the adult mouse brain. Towards this, we injected a Cre-dependent mVenus adeno-associated virus (AAV-DIO-mVenus) and a Flp-dependent mRuby2 adeno-associated virus (AAV-fDIO-mRuby2) into the LSN of adult ChAT-Cre; vGAT-Flp mice (Fig. 1D). We identified a large number mVenus-positive cholinergic neurons, mRuby2-positive GABAergic neurons, and dually labeled co-expressing neurons (Fig. 1E-F). These findings confirmed the existence of ChAT/vGAT co-expressing neurons within the adult LSN and suggested that labeled cells identified via genetic reporter expression were not just a remnant of neurotransmitter switching during development.

### FISH and antibody-based immunohistochemistry detect co-expressing LSN neurons

**Fig. 2:**
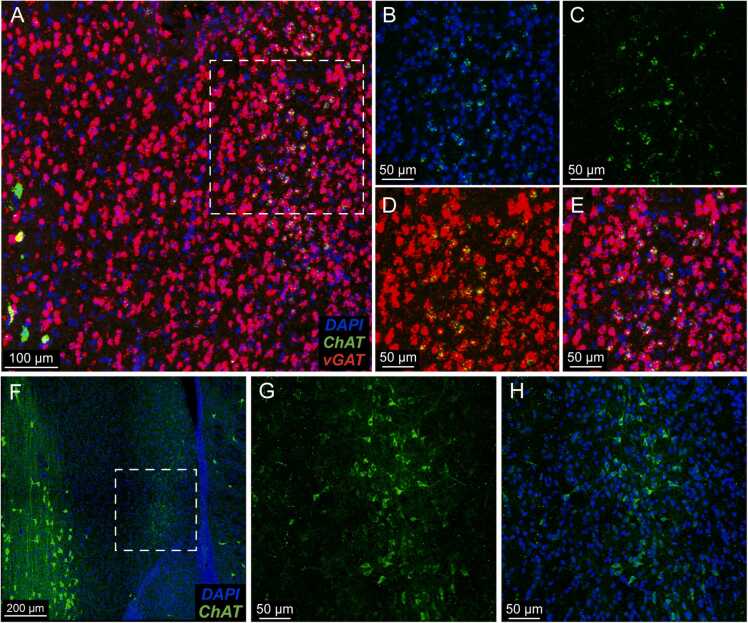
FISH and antibody staining reflect ChAT/vGAT co-expressing population of neurons within the LSN. (A) FISH stain of the LSN with (B) magnified image of region shown within the dotted square, reflecting expression of DAPI and ChAT, (C) ChAT alone, (D) vGAT and ChAT, and (E) merged signal with co-expression of vGAT and ChAT within the same neurons. (F) IHC stain after primary anti-ChAT antibody incubation with (G) magnified image of region shown within the dotted square, reflecting low but clearly appreciable ChAT expression within the lateral LSN. (H) Magnified image of merged ChAT and DAPI images.

Noting the paucity of literature describing either cholinergic neurons or ChAT/vGAT co-expressing neurons within the LSN (Ichikawa et al., 1997, Rossi et al., 2011, Saunders et al., 2015a), we performed fluorescent in situ hybridization (FISH) to label ChAT and vGAT transcripts throughout the basal forebrain. Consistent with what we observed via genomic and viral labeling experiments, indeed we identified ChAT/vGAT co-expressing neurons in the LSN (Fig. 2A-B). ChAT expression in these neurons appeared lower when compared to nearby ChAT positive neurons in the vertical domain of the diagonal band of Broca (VDB) (Fig. 2A, bottom left). Indeed, when examining data from the Allen Brain Atlas, we found a similar pattern of low ChAT expression within the vGAT-rich region of the LSN (Supplementary Fig. 2) (Lein et al., 2007). Importantly, we found ChAT expression only within the lateral portion of the LSN, juxtaposing the lateral ventricle (+1.1 to +0.38 mm from Bregma). This excluded the most anterior and posterior portions of the LSN, which extends from Bregma + 1.7 to − 0.45 mm (Franklin and Paxinos, 2008). To further characterize these neurons, we performed immunohistochemistry (IHC) to stain for ChAT expression, which allowed us to directly observe ChAT-positive LSN neurons. These neurons had less fluorescent intensity when compared to ChAT-positive neurons within the VDB (Fig. 2C, bottom left), and consistent with FISH data, we only identified these neurons within the lateral LSN (Fig. 2C-D).

### Number of co-expressing LSN neurons increases with time

**Fig. 3:**
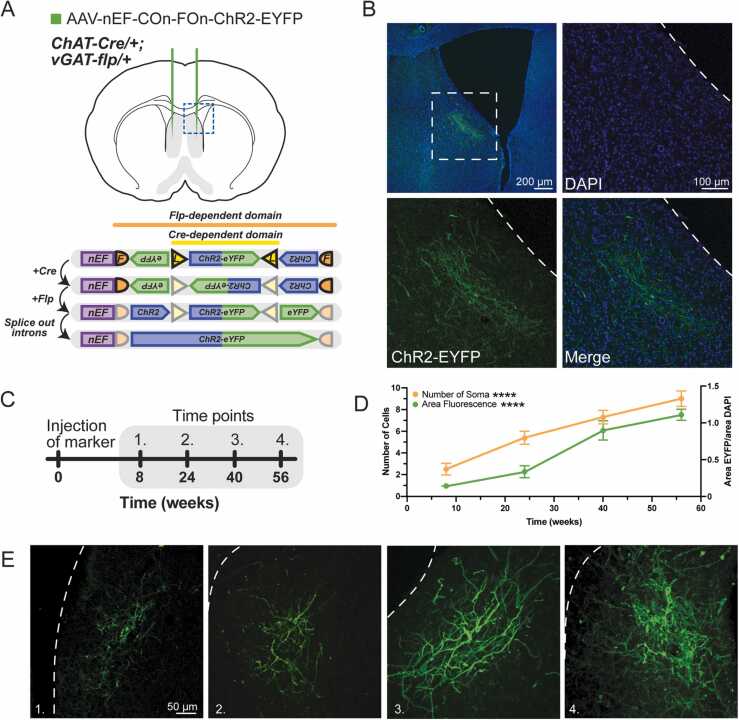
Length of marker incubation increases the number of labeled co-expressing neurons within the LSN. (A) Top: Viral labeling of co-expressing neurons in the LSN. Bottom: AAV-nEF-COn-FOn-ChR2-EYFP mechanism of selective expression in Cre/Flp-positive neurons. (B) LSN neurons expressing ChR2-EYFP. (C) Time course for length of viral incubation. (D) EYFP expression graphed over time. n = 30 images (bilateral LSN) quantified from 3 animals. (E) Images of the LSN at each time point, corresponding to the graphed fluorescence.

Co-expressing neurons have been shown to exist in a transcriptionally dynamic state, in which one or more of the neurotransmitter-specific markers changes expression over time (Spitzer, 2015, Dulcis et al., 2017, Li et al., 2020). This type of expression profile provides insight into the function of the co-transmitting neuron, while also providing a potential explanation as to why these populations have not received previous attention. To address the possibility of dynamic ChAT and/or vGAT expression within lateral LSN neurons, we leveraged a Cre- and Flp-dependent viral channelrhodopsin-EYFP reporter (AAV-nEF-COn-FOn-ChR2-EYFP) (Fenno et al., 2014) to label both the cell bodies and membranes of ChAT-Cre and vGAT-Flp co-expressing neurons within the LSN (Fig. 3A). We selected a membrane bound reporter to increase the detection of labeled neuronal surface area, thereby allowing us to measure a greater dynamic range in reporter expression. Using this vector, we confirmed potent expression of the conditional AAV reporter 8 wks following injection (Fig. 3B). After this period, we harvested tissue and imaged samples at three additional time points 16 wks apart (Fig. 3C). We hypothesized that if neurotransmitter switching was occurring, by allowing longer times for the viral label to incubate, we would detect increased numbers of co-expressing neurons. Indeed, through this approach we found increased expression of the Cre- and Flp-dependent viral label over time, exhibiting both increased signal intensity and increased number of EYFP-positive cell bodies (Fig. 3D). To ensure that the injected viral reporter marked only cells that expressed both Cre and Flp, we also injected this same construct into control wildtype animals and incubated the marker for 24 weeks. After this incubation period, we found no expression of the reporter (Supplementary Fig. 3), suggesting that increased viral reporter expression observed in LSN neurons (Fig. 3E) was due to ChAT and vGAT expression dynamically changing within that population over time. To determine whether the increased co-expression of ChAT and vGAT was due to aging, we performed FISH analysis in wildtype mice at 24 and 44 weeks of age, comparing the number of ChAT/vGAT co-positive neurons in the LSN at these two ages. This analysis found no difference in the number of ChAT/vGAT co-expressing neurons between the two groups (Supplementary Fig. 4).

### LSN neurons co-release ACh and GABA onto glutamatergic LSN neurons

**Fig. 4:**
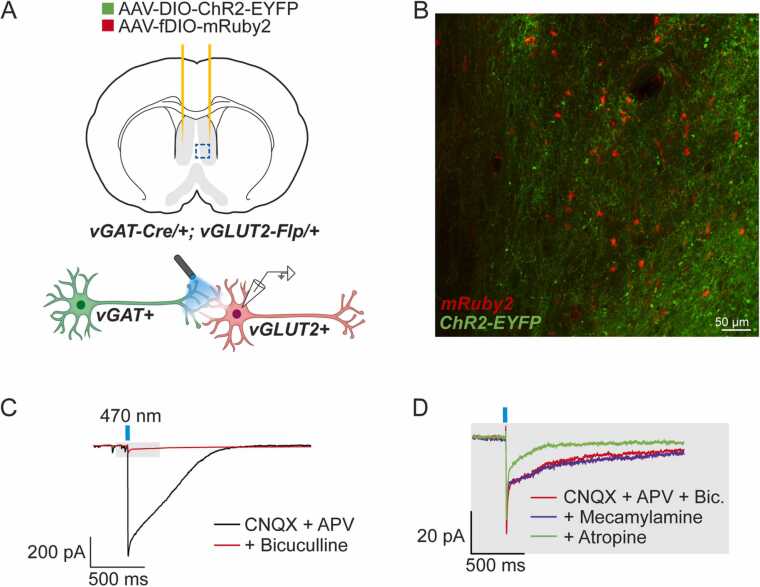
LSN co-expressing neurons co-release ACh and GABA onto vGlut2-positive LSN neurons. (A) Photostimulation of ChR2-expressing vGAT-positive neurons while recording from mRuby2-expressing vGlut2-positive neurons in the LSN. (B) Confocal image of LSN with labeled channelrhodopsin-expressing vGAT-Cre-positive neurons (green) and vGlut2-Flp-positive neurons (red). (C) Recording of IPSCs in the presence of glutamatergic and GABAergic blockers. (D) Magnification of grey box in (C), IPSCs are resistant to bath application of mecamylamine, but sensitive to bath application of atropine.

To validate the functional expression of ACh and GABA in LSN neurons, we next tested whether the identified co-expressing LSN neurons release both ACh and GABA onto postsynaptic targets via electrophysiological recordings. Due to the large number of GABAergic neurons within the LSN (Fig. 2A,D, Supplementary Fig. 2B) (Lein et al., 2007, Zhao et al., 2013), we targeted the light-gated ion channel, channelrhodopsin (ChR2) to vGAT^LSN^ neurons, and subsequently measured for cholinergic and GABAergic co-transmission onto postsynaptic targets. Assuming that subpopulations of vGAT^LSN^ neurons would have connections within the LSN, we labeled local glutamatergic neurons by injecting a combination of Cre-dependent ChR2 (AAV-DIO-ChR2-EYFP) and Flp-dependent mRuby2 (AAV-fDIO-mRuby2) viruses into the LSN of vGAT-Cre; vGlut2-Flp (JAX Stock No. 030212) mice (Fig. 4A-B). While performing whole-cell recordings from red labeled vGlut2^LSN^ neurons, and simultaneously photostimulating vGAT^LSN^ inputs in the presence of both TTX and 4-AP, we found that 100% of patched vGlut2^LSN^ neurons received fast, monosynaptic, GABAergic currents (n = 15/15 cells), and 20% of these vGlut2^LSN^ neurons also received small (5–20 pA) metabotropic, cholinergic currents (n = 3/15 cells), as evidenced by light-evoked currents that were blocked by the application of the mAChR-specific antagonist atropine (Fig. 4B,C). Application of the nicotinic receptor antagonist mecamylamine did not affect photo-evoked currents.

Additionally, we injected a mixture of AAV-DIO-ChR2-EYFP and AAV-fDIO-mRuby2 viruses into the LSN of ChAT-Cre; vGlut2-Flp mice, to record cholinergic and GABAergic release from ChAT-Cre-positive LSN neurons onto vGlut2-Flp-positive LSN neurons. Recording 17 cells from 4 separate animals, we found neither cholinergic, nor GABAergic responses in mRuby2-positive cells (data not shown), suggesting the numbers of co-transmitting neurons is likely small.

Together, these data show that a subpopulation of vGAT^LSN^ neurons indeed release both GABA and ACh onto postsynaptic target neurons, demonstrating that ACh/GABA co-transmission occurs within the LSN.

## Discussion

Understanding how neurons function together to manifest emergent circuit properties of the brain remains largely an ongoing challenge. This is due, at least in part, to our lack of a full mechanistic understanding of the detailed modes of neurotransmission between diverse populations of brain cells. Recent advances in neural circuit research have uncovered an incredible diversity of cell types and synaptic connections throughout the brain (Saunders et al., 2018, Huang et al., 2020). However, many of these advances account only for the expression and connectivity patterns of neural circuits statically in time and are largely unable to define how neurons within these circuits dynamically change.

Evidence demonstrating circuit and organism-level transcriptional changes throughout development, and in response to diverse environmental stimuli, illustrates the necessity of understanding how neural circuit activity changes in different contexts. Assays including in situ staining techniques and sequencing approaches account for the state of neuronal populations only statically due to the short temporal nature of these assays. However, genetic and conditional viral labeling techniques allow for the incubation of a conditional marker within defined populations of neurons, thereby labeling any neuron that satisfies the condition of the reporter over the course of incubation. By combining both short- and long-timescale vital labeling techniques we have uncovered a population of ACh/GABA co-transmitting neurons within the lateral LSN, that changes the expression of neurotransmission related genes over time. We have found that ChAT expression in this neuronal population appears low using FISH and IHC. This may explain why the presence of these neurons has been largely overlooked.

To explore whether ChAT/vGAT co-expressing neurons within the LSN exhibited dynamic transcriptional regulation, we leveraged a conditional viral reporter that expressed a membrane bound label only in the presence of both Cre and Flp. The observed increase in expression of this fluorescent reporter is congruent with ChAT expression in vGAT^LSN^ neurons, which changes over time. Here we propose that vGAT-Flp-positive neurons lowly express ChAT-Cre at distinct periods throughout time. This low expression of Cre, in the presence of vGAT-driven Flp, is sufficient to permanently activate the Cre and Flp dependent conditional reporter, resulting in the increased labeling seen at later time points. The increased number of EYFP-positive cell bodies suggests that an increasing number of vGAT^LSN^ neurons are turning on expression of the ChAT-Cre allele at some point in time. This experimental approach provides no evidence of cessation of ChAT expression, as the EYFP marker is permanently expressed once it has been activated in these neurons (Fig. 3C-E). However, these findings illustrate a dynamic expression of ChAT in vGAT^LSN^ neurons. Importantly, though this dynamic expression in the context of neural co-transmission demonstrates features of neurotransmitter switching, this observation does not strictly meet the criteria to be considered a *bona fide* neurotransmitter switching event (Spitzer, 2017), as there is no evidence of loss of vGAT expression in these neurons. Additionally, many of the reported cases of neurotransmitter switching have been associated with an inciting stimulus that drives the observed transcriptional changes. At the time of this report, we have not identified a conserved stimulus, if any, that is able to selectively drive the observed expression changes. However, based on our findings, we reason that whatever factor causes the changes in expression is experienced by the mice in an activity-dependent manner over time in their home cages.

The function of such dynamic transcriptional changes is not obvious. That ChAT is responsible for the synthesis of ACh suggests that these observed transcriptional changes might give neurons the ability to release ACh. Indeed, we find that, along with GABA, vGAT^LSN^ neurons release ACh onto local glutamatergic neurons, implying that the observed transient expression of ChAT is at least sufficient to drive ACh production and release onto a subset of downstream circuitry. During these experiments we observed a residual bicuculline-, mecamylamine- and atropine-resistant current. Upon further analysis, we found that this current has a reversal potential near − 55 mV, suggesting that it is likely a chloride-linked current. However, the current is not sensitive to bath application of the GABA_C_ receptor blocker, TPMPA at 20 μM (data not shown). Thus, it is possible that this current is the result of incomplete inhibition by the competitive antagonist bicuculline. However, further experimentation is necessary to confirm this hypothesis. That we observed evidence of co-transmission postsynaptically in only a small fraction of the vGlut2^LSN^ neurons likely reflects the rare and transient expression of ChAT in the vGAT^LSN^ population. However, this might also reflect variation in the expression of muscarinic and nicotinic receptors by vGlut2^LSN^ neurons. Dynamic changes in postsynaptic receptor expression that pair with pre-synaptic neurotransmitter expression changes have been described in other regions of the rodent brain (Kotak et al., 1998, Korada and Schwartz, 1999). Future work will be necessary to determine the receptor expression profile of vGlut2^LSN^ neurons, as well as the frequency and magnitude of ChAT expression changes in vGAT^LSN^ neurons over time, and to what stimulus.

The low intensity of ChAT staining within LSN neurons using traditional staining techniques is likely the reason why this population has not garnered previous attention. This could explain why leveraging conditional genetic methods to label these neurons provides better visualization, as the high recombination efficiency of Cre, even at low concentrations, allows for strong expression of the conditional reporter. However, this hypothesis cannot fully explain the increased signal observed when incubating a Cre- and Flp-dependent marker over longer periods of time. Rather, this observed increase in the intensity of signal and in the number of EYFP-positive neurons is more consistent with dynamic changes in ChAT expression within the vGAT^LSN^ population. Moreover, electrophysiological evidence suggests that the observed ChAT-expression is sufficient for the release of ACh from this neuronal population.

We identified local vGlut2^LSN^ neurons as a postsynaptic target of both ACh and GABA release from co-transmitting LSN neurons, and the muscarinic nature of the cholinergic component of this co-transmission suggests a role for this concerted release in regulating the activity of postsynaptic targets. In such a model, muscarinic signaling may modulate the activity of postsynaptic targets in conjunction with synaptic GABAergic inhibition. Similar synergistic regulation has been reported in other areas of the brain, including the olfactory bulb (Pressler et al., 2007, Zhan et al., 2013, Smith et al., 2015), and striatum (Suzuki and Momiyama, 2021). Alternatively, ACh release may be exerting a presynaptic effect on vGAT^LSN^ neurons similar to what has been previously reported (Grilli et al., 2009, Nakamura and Jang, 2012). However, future experimentation is needed to assess these mechanistic possibilities.

Finally, we report that transcriptional changes in adult mice, previously known to be the primary mechanism driving neurotransmitter switching, also constitutes a candidate mechanism to facilitate neural co-transmission. Attention in these two fields of study – neurotransmitter switching and neural co-transmission - is rapidly growing. However, to date, only a few examples have linked the two neural phenotypes (Furshpan et al., 1976, Spitzer, 2015). This work provides further rationale to examine both neural states together. Whether previously reported examples of neural co-transmission reflect neurons in a transient transcriptional state needs to be more closely examined. The results of these future experiments will help us better understand both neural circuit biology, and the plasticity mechanisms that govern the astounding dynamics that define neuronal circuits.

## Funding sources

This work was supported by the 10.13039/100000968American Heart Association (AHA 20PRE35040011 to PH), the McNair Medical Institute, and the 10.13039/100000002National Institutes of Health (R01 EB027145-01A1/04 and R01 NS078294-06A1 to BA). This project was also supported in part by the RNA In Situ Hybridization Core facility at Baylor College of Medicine, which is supported by a Shared Instrumentation grant from the NIH (1S10OD016167) and the NIH IDDRC grant P50 HD103555 from the Eunice Kennedy Shriver National Institute of Child Health & Human Development. The content is solely the responsibility of the authors and does not necessarily represent the official views of the Eunice Kennedy Shriver National Institute of Child Health & Human Development or the National Institutes of Health.

## CRediT authorship contribution statement

**Patrick J. Hunt:** Conceptualization, Methodology, Investigation, Analysis, Writing. **Mikhail Kochukov:** Investigation, Analysis. **Brandon T. Pekarek:** Investigation, Methodology, Data curation, Writing revision. **Benjamin D. W. Belfort:** Investigation, Methodology, Data curation, Writing revision. **Juan Romero:** Investigation, Methodology, Data curation, Writing revision. **Jessica L. Swanson:** Investigation, Methodology, Data curation, Writing revision. **Benjamin R. Arenkiel:** Supervision, Conceptualization, Resources, Writing.

## References

[bib1] Albert D.J., Richmond S.E. (1976). Hyperreactivity and aggressiveness following infusion of local anesthetic into the lateral septum or surrounding structures. Behav. Biol..

[bib2] Armstrong D.M., Saper C.B., Levey A.I., Wainer B.H., Terry R.D. (1983). Distribution of cholinergic neurons in rat brain: demonstrated by the immunocytochemical localization of choline acetyltransferase. J. Comp. Neurol..

[bib3] Case D.T., Burton S.D., Gedeon J.Y., Williams S.P.G., Urban N.N., Seal R.P. (2017). Layer- and cell type-selective co-transmission by a basal forebrain cholinergic projection to the olfactory bulb. Nat. Commun..

[bib4] Chaudhry F.A., Reimer R.J., Bellocchio E.E., Danbolt N.C., Osen K.K., Edwards R.H., Storm-Mathisen J. (1998). The vesicular GABA transporter, VGAT, localizes to synaptic vesicles in sets of glycinergic as well as GABAergic neurons. J. Neurosci..

[bib5] Dulcis D., Jamshidi P., Leutgeb S., Spitzer N.C. (2013). Neurotransmitter switching in the adult brain regulates behavior. Science.

[bib6] Dulcis D., Lippi G., Stark C.J., Do L.H., Berg D.K., Spitzer N.C. (2017). Neurotransmitter switching regulated by miRNAs controls changes in social preference. Neuron.

[bib7] Fenno L.E. (2014). Targeting cells with single vectors using multiple-feature Boolean logic. Nat. Methods.

[bib8] Franklin K., Paxinos G. (2008). The Mouse Brain in Stereotaxic Coordinates.

[bib9] Furshpan E.J., MacLeish P.R., O’Lague P.H., Potter D.D. (1976). Chemical transmission between rat sympathetic neurons and cardiac myocytes developing in microcultures: evidence for cholinergic, adrenergic, and dual function neurons. Proc. Natl. Acad. Sci. U.S.A..

[bib10] Gizowski C., Bourque C.W. (2020). Sodium regulates clock time and output via an excitatory GABAergic pathway. Nature.

[bib11] Granger A.J., Mulder N., Saunders A., Sabatini B.L. (2016). Cotransmission of acetylcholine and GABA. Neuropharmacology.

[bib12] Granger A.J., Wang W., Robertson K., El-Rifai M., Zanello A.F., Bistrong K., Saunders A., Chow B.W., Nuñez V., Turrero García M., Harwell C.C., Gu C., Sabatini B.L. (2020). Cortical ChAT+ neurons co-transmit acetylcholine and GABA in a target- and brain-region-specific manner. Elife.

[bib13] Graybuck L.T. (2021). Enhancer viruses for combinatorial cell-subclass-specific labeling. Neuron.

[bib14] Grilli M., Zappettini S., Raiteri L., Marchi M. (2009). Nicotinic and muscarinic cholinergic receptors coexist on GABAergic nerve endings in the mouse striatum and interact in modulating GABA release. Neuropharmacology.

[bib15] Huang L., Kebschull J.M., Fürth D., Musall S., Kaufman M.T., Churchland A.K., Zador A.M. (2020). BRICseq bridges brain-wide interregional connectivity to neural activity and gene expression in single animals. Cell.

[bib16] Ichikawa T., Ajiki K., Matsuura J., Misawa H. (1997). Localization of two cholinergic markers, choline acetyltransferase and vesicular acetylcholine transporter in the central nervous system of the rat: In situ hybridization histochemistry and immunohistochemistry. J. Chem. Neuroanat..

[bib17] Kocsis K., Kiss J., Csáki A., Halász B. (2003). Location of putative glutamatergic neurons projecting to the medial preoptic area of the rat hypothalamus. Brain Res. Bull..

[bib18] Korada S., Schwartz I.R. (1999). Development of GABA, glycine, and their receptors in the auditory brainstem of gerbil: A light and electron microscopic study. J. Comp. Neurol..

[bib19] Kotak V.C., Korada S., Schwartz I.R., Sanes D.H. (1998). A developmental shift from GABAergic to glycinergic transmission in the central auditory system. J. Neurosci..

[bib20] Lee S., Kim K., Zhou Z.J. (2010). Role of ACh-GABA Cotransmission in Detecting Image Motion and Motion Direction. Neuron.

[bib21] Lein E.S. (2007). Genome-wide atlas of gene expression in the adult mouse brain. Nature.

[bib22] Leroy F., Park J., Asok A., Brann D.H., Meira T., Boyle L.M., Buss E.W., Kandel E.R., Siegelbaum S.A. (2018). A circuit from hippocampal CA2 to lateral septum disinhibits social aggression. Nature.

[bib23] Li H., Quan, Spitzer N.C. (2020). Exercise enhances motor skill learning by neurotransmitter switching in the adult midbrain. Nat. Commun..

[bib24] Li H.Q., Pratelli M., Godavarthi S., Zambetti S., Spitzer N.C. (2020). Decoding neurotransmitter switching: the road forward. J. Neurosci..

[bib25] Lin W., McKinney K., Liu L., Lakhlani S., Jennes L. (2003). Distribution of vesicular glutamate transporter-2 messenger ribonucleic acid and protein in the septum-hypothalamus of the rat. Endocrinology.

[bib26] Lusk S.J., McKinney A., Hunt P.J., Fahey P.G., Patel J., Chang A., Sun J.J., Martinez V.K., Zhu P.J., Egbert J.R., Allen G., Jiang X., Arenkiel B.R., Tolias A.S., Costa-Mattioli M., Ray R.S. (2022). A CRISPR toolbox for generating intersectional genetic mouse models for functional, molecular, and anatomical circuit mapping. BMC Biol..

[bib27] Nabekura J., Katsurabayashi S., Kakazu Y., Shibata S., Matsubara A., Jinno S., Mizoguchi Y., Sasaki A., Ishibashi H. (2004). Developmental switch from GABA to glycine release in single central synaptic terminals. Nat. Neurosci..

[bib28] Nakamura M., Jang I.S. (2012). Muscarinic M 4 receptors regulate GABAergic transmission in rat tuberomammillary nucleus neurons. Neuropharmacology.

[bib29] Pressler R.T., Inoue T., Strowbridge B.W. (2007). Muscarinic receptor activation modulates granule cell excitability and potentiates inhibition onto mitral cells in the rat olfactory bulb. J. Neurosci..

[bib30] Root D.H., Mejias-Aponte C.A., Zhang S., Wang H.L., Hoffman A.F., Lupica C.R., Morales M. (2014). Single rodent mesohabenular axons release glutamate and GABA. Nat. Neurosci..

[bib31] Rossi J., Balthasar N., Olson D., Scott M., Berglund E., Lee C.E., Choi M.J., Lauzon D., Lowell B.B., Elmquist J.K. (2011). Melanocortin-4 receptors expressed by cholinergic neurons regulate energy balance and glucose homeostasis. Cell Metab..

[bib32] Saunders A., Granger A.J., Sabatini B.L. (2015). Corelease of acetylcholine and GABA from cholinergic forebrain neurons. Elife.

[bib33] Saunders A., Macosko E.Z., Wysoker A., Goldman M., Krienen F.M., de Rivera H., Bien E., Baum M., Bortolin L., Wang S., Goeva A., Nemesh J., Kamitaki N., Brumbaugh S., Kulp D., McCarroll S.A. (2018). Molecular diversity and specializations among the cells of the adult mouse brain. Cell.

[bib34] Saunders A., Oldenburg I. a, Berezovskii V.K., Johnson C. a, Kingery N.D., Elliott H.L., Xie T., Gerfen C.R., Sabatini B.L. (2015). A direct GABAergic output from the basal ganglia to frontal cortex. Nature.

[bib35] Shabel S.J., Proulx C.D., Piriz J., Malinow R. (2014). Mood regulation. GABA/glutamate co-release controls habenula output and is modified by antidepressant treatment. Science.

[bib36] Sheehan T.P., Chambers R.A., Russell D.S. (2004). Regulation of affect by the lateral septum: Implications for neuropsychiatry. Brain Res. Rev..

[bib37] Singewald G.M., Rjabokon A., Singewald N., Ebner K. (2011). The modulatory role of the lateral septum on neuroendocrine and behavioral stress responses. Neuropsychopharmacology.

[bib38] Smith R.S., Hu R., DeSouza A., Eberly C.L., Krahe K., Chan W., Araneda R.C. (2015). Differential muscarinic modulation in the olfactory bulb. J. Neurosci..

[bib39] Spitzer N.C. (2015). Neurotransmitter switching? No surprise. Neuron.

[bib40] Spitzer N.C. (2017). Neurotransmitter switching in the developing and adult brain. Annu. Rev. Neurosci..

[bib41] Suzuki E., Momiyama T. (2021). M1 muscarinic acetylcholine receptor-mediated inhibition of GABA release from striatal medium spiny neurons onto cholinergic interneurons. Eur. J. Neurosci..

[bib42] Sweeney P., Yang Y. (2015). An excitatory ventral hippocampus to lateral septum circuit that suppresses feeding. Nat. Commun..

[bib43] Sweeney P., Yang Y. (2016). An inhibitory septum to lateral hypothalamus circuit that suppresses feeding. J. Neurosci..

[bib44] Takács V.T., Cserép C., Schlingloff D., Pósfai B., Szőnyi A., Sos K.E., Környei Z., Dénes Á., Gulyás A.I., Freund T.F., Nyiri G. (2018). Co-transmission of acetylcholine and GABA regulates hippocampal states. Nat. Commun..

[bib45] Tatti R., Haley M.S., Swanson O.K., Tselha T., Maffei A. (2017). Neurophysiology and regulation of the balance between excitation and inhibition in neocortical circuits. Biol. Psychiatry.

[bib46] van der Veldt S., Etter G., Mosser C.-A., Manseau F., Williams S. (2021) Conjunctive spatial and self-motion codes are topographically organized in the GABAergic cells of the lateral septum. 10.1371/journal.pbio.3001383 PMC8432898 34460812

[bib47] Verney C., Gaspar P., Alvarez C., Berger B. (1987). Postnatal sequential development of dopaminergic and enkephalinergic perineuronal formations in the lateral septal nucleus of the rat correlated with local neuronal maturation. Anat. Embryol..

[bib48] Vong L., Ye C., Yang Z., Choi B., Chua S., Lowell B.B. (2011). Leptin action on GABAergic neurons prevents obesity and reduces inhibitory tone to POMC neurons. Neuron.

[bib49] Yaylaoglu M.B., Titmus A., Visel A., Alvarez-Bolado G., Thaller C., Eichele G. (2005). Comprehensive expression atlas of fibroblast growth factors and their receptors generated by a novel robotic in situ hybridization platform. Dev. Dyn..

[bib50] Zhan X., Yin P., Heinbockel T. (2013). The basal forebrain modulates spontaneous activity of principal cells in the main olfactory bulb of anesthetized mice. Front. Neural Circuits.

[bib51] Zhao C., Eisinger B., Gammie S.C. (2013). Characterization of GABAergic neurons in the mouse lateral septum: a double fluorescence in situ hybridization and immunohistochemical study using tyramide signal amplification. PLoS One.

