# Supplementary figures and images for: Co-transmitting neurons in the lateral septal nucleus exhibit features of neurotransmitter switching
Source: IBRO Neurosci Rep. 2022 May 12;12:390–8. doi: 10.1016/j.ibneur.2022.05.003 (PMC9121281; doi:10.1016/j.ibneur.2022.05.003)

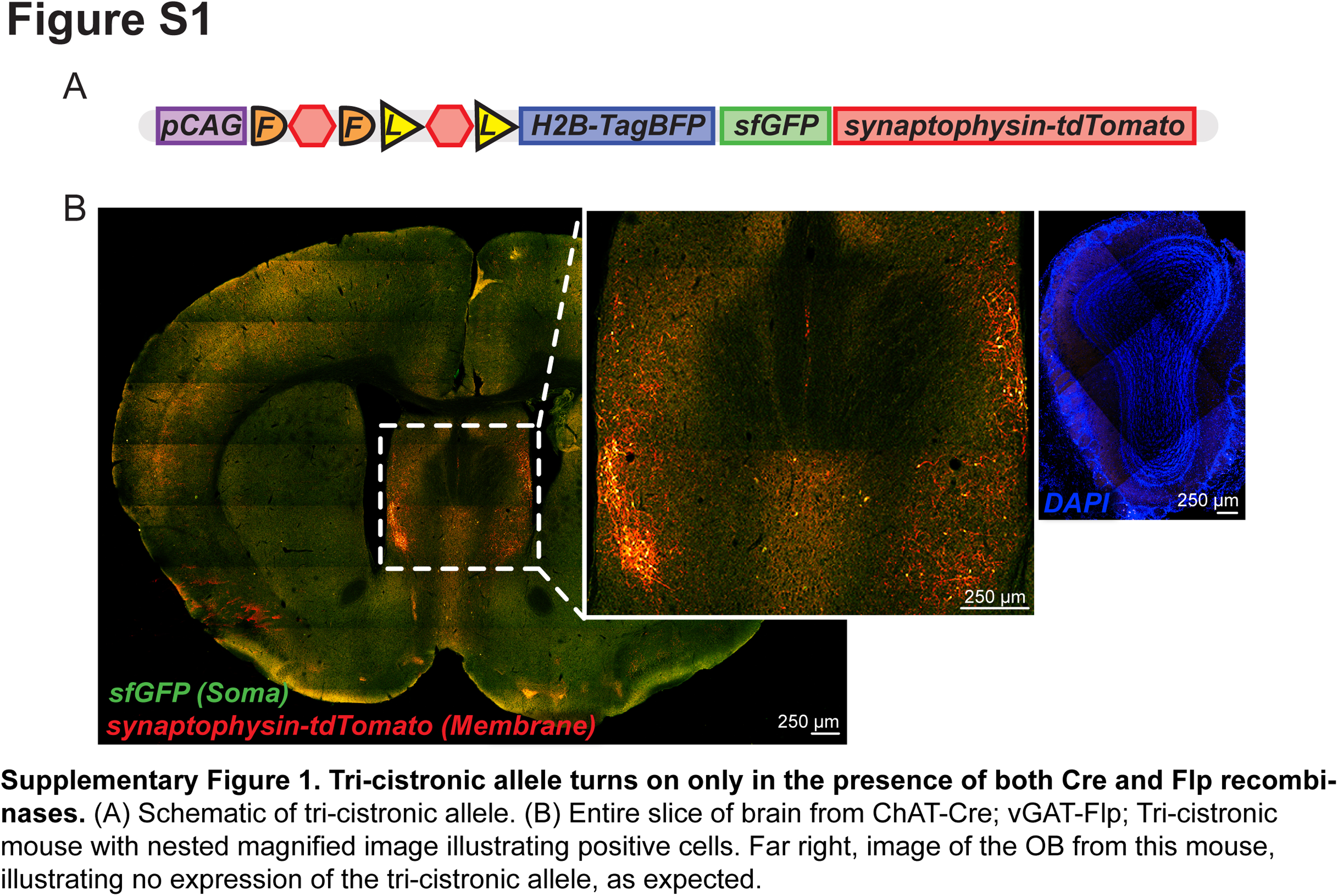

Supplement: Supplementary file 1 — Supplementary material [file mmc1.jpg]

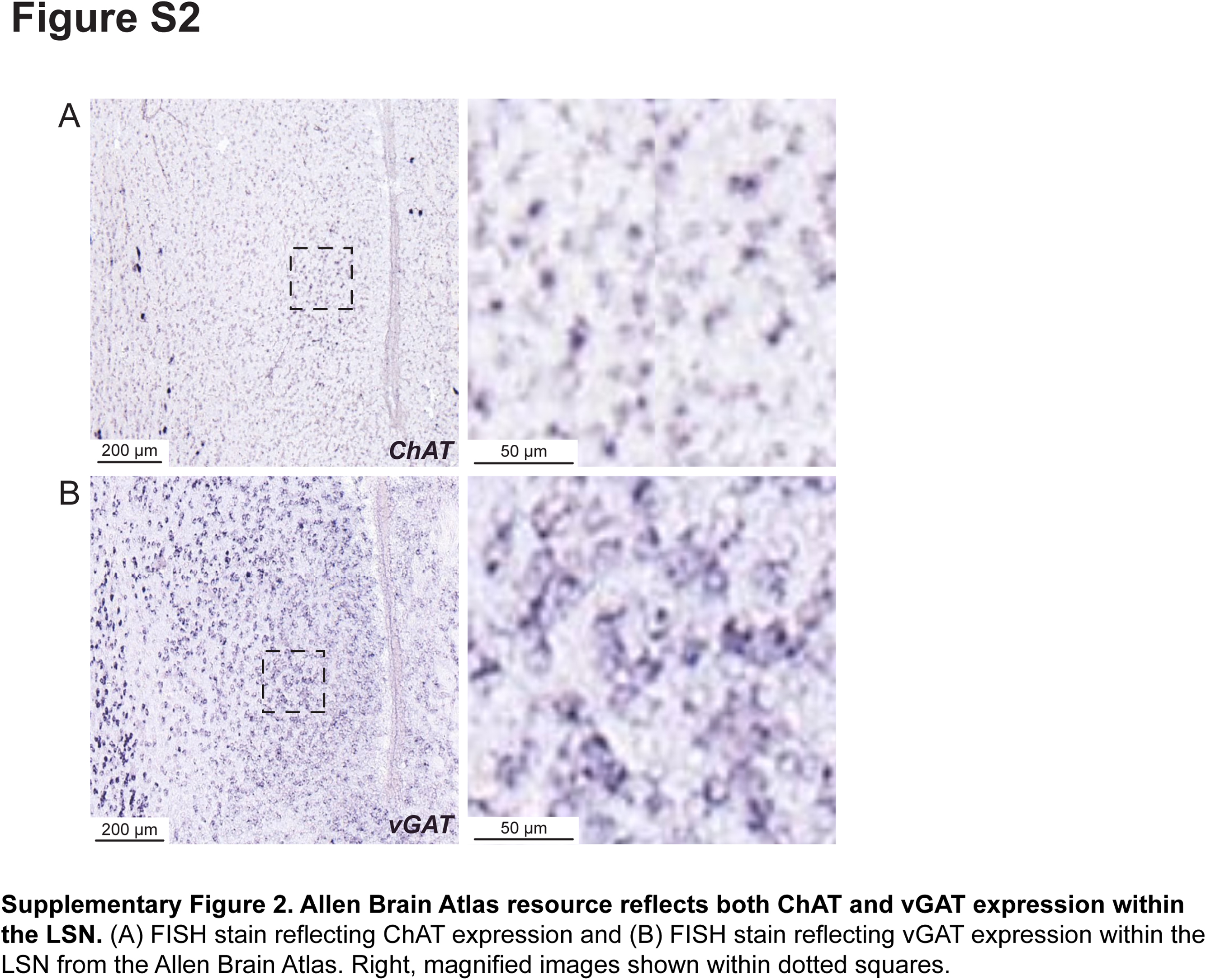

Supplement: Supplementary file 2 — Supplementary material [file mmc2.jpg]

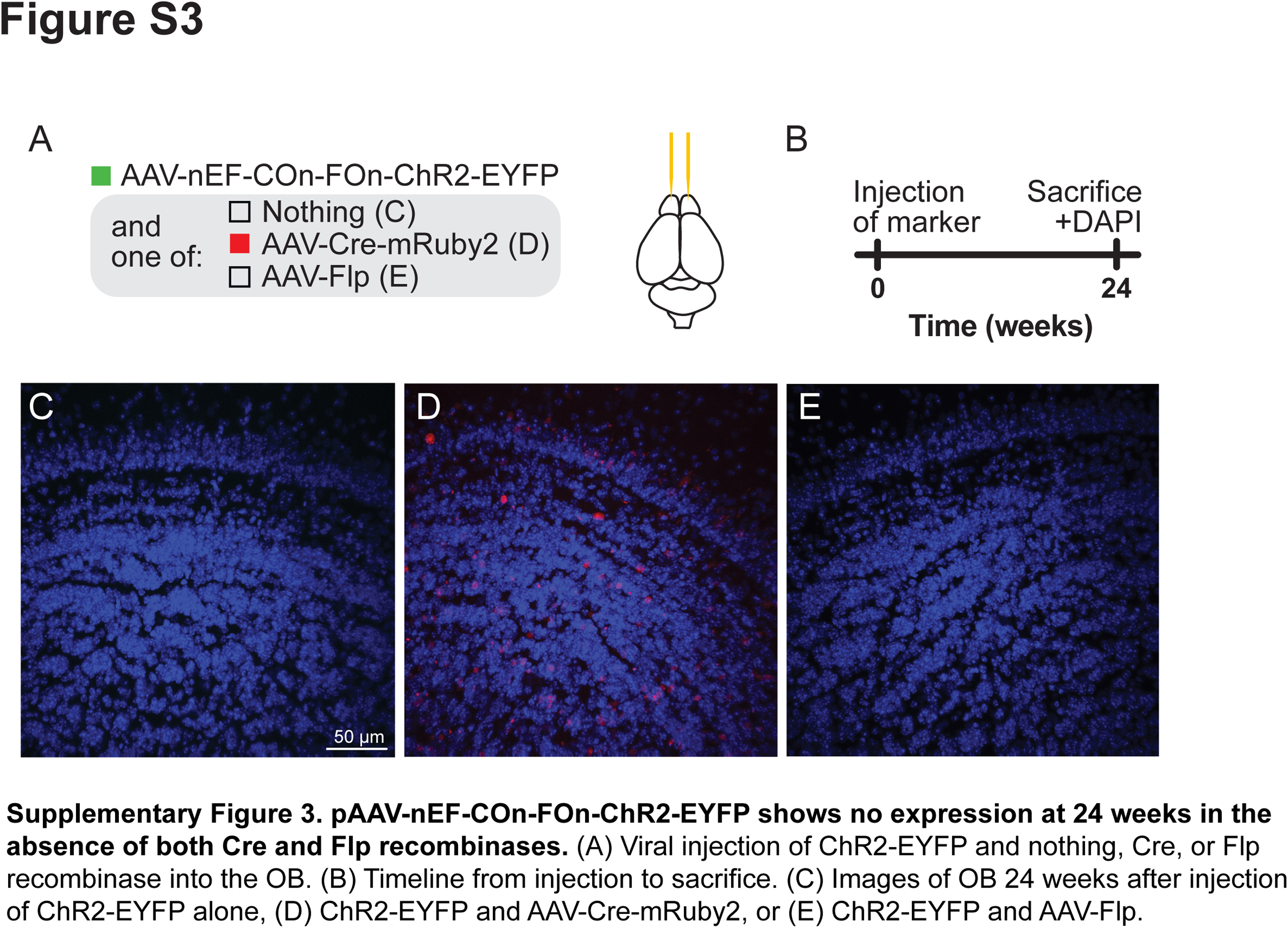

Supplement: Supplementary file 3 — Supplementary material [file mmc3.jpg]

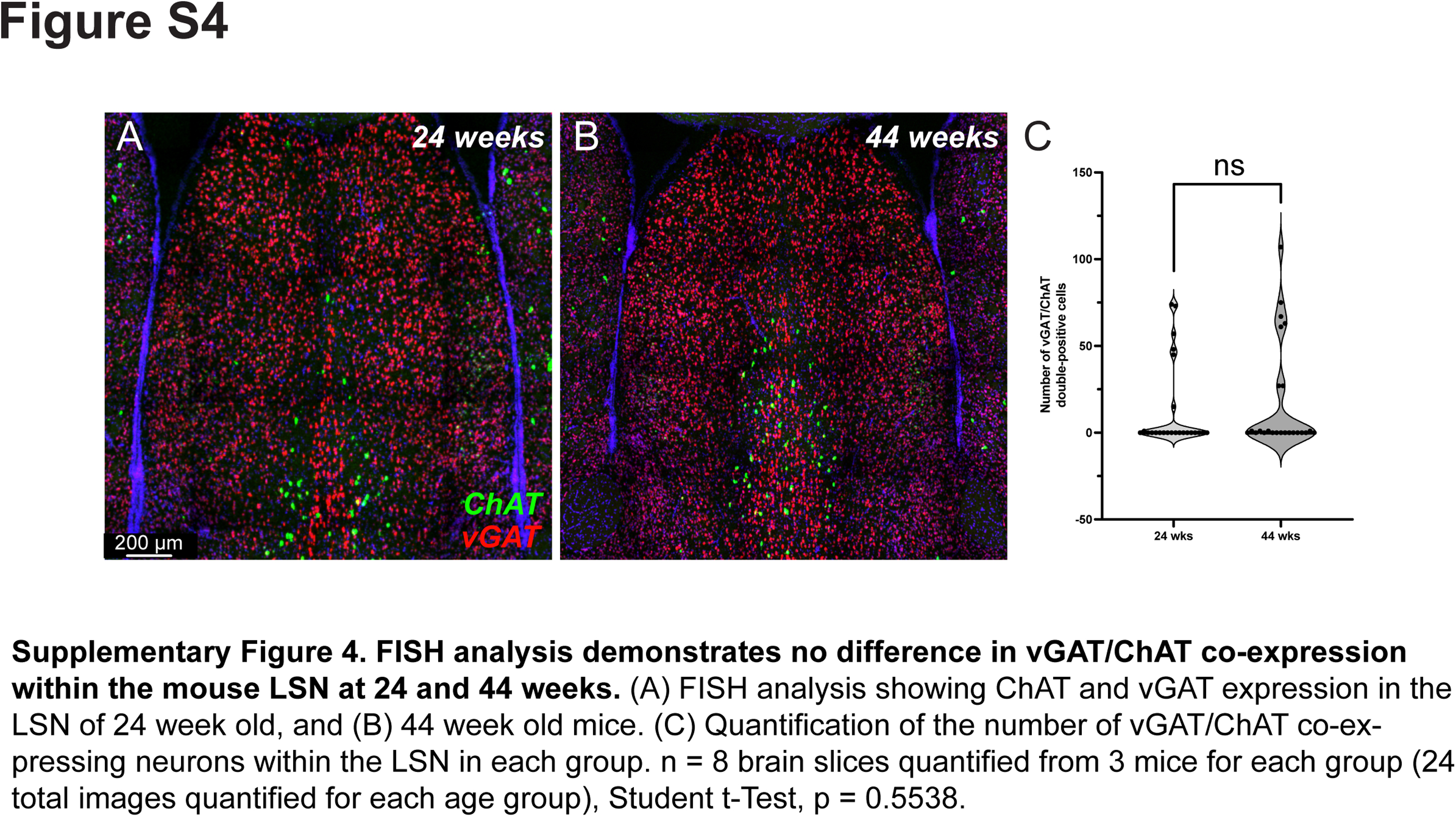

Supplement: Supplementary file 4 — Supplementary material [file mmc4.jpg]
